# Stented endoscopic third ventriculostomy: technique, safety, and indications—a multicenter multinational study

**DOI:** 10.1007/s00381-024-06566-7

**Published:** 2024-08-05

**Authors:** Lee Azolai, Valentina Pennacchietti, Matthias Schulz, Henry W. S. Schroeder, Petr Vacek, Shlomi Constantini, Lidor Bitan, Jonathan Roth, Ulrich-Wilhelm Thomale

**Affiliations:** 1 Departments of Neurosurgery and Pediatric Neurosurgery, Dana Children’s Hospital, Tel Aviv Medical Center, Tel Aviv University, Tel Aviv, Israel; 2 Pediatric Neurosurgery, Charité Universitaetsmedizin Berlin, Berlin, Germany; 3 Department of Neurosurgery, University Medicine Greifswald, Greifswald, Germany; 4 Department of Neurosurgery, Faculty of Medicine in Plzeň, University Hospital, Charles University, Pilsen, Czech Republic

**Keywords:** Stent, Endoscopic third ventriculostomy, Hydrocephalus, Ommaya, Scarring

## Abstract

**Purpose:**

Endoscopic third ventriculostomy (ETV) is an effective treatment for obstructive hydrocephalus. Secondary stoma closure may be life threatening and is the most common reason for late ETV failure, mostly secondary to local scarring. Local stents intended to maintain patency are rarely used. In this study, we summarize our experience using stented ETV (sETV), efficacy, and safety.

**Material and methods:**

Data was retrospectively collected from all consecutive patients who underwent ETV with stenting at four centers. Collected data included indications for using sETV, hydrocephalic history, surgical technique, outcomes, and complications.

**Results:**

Sixty-seven cases were included. Forty had a primary sETV, and 27 had a secondary sETV (following a prior shunt, ETV, or both). The average age during surgery was 22 years. Main indications for sETV included an adjacent tumor (*n* = 15), thick or redundant tuber cinereum (*n* = 24), and prior ETV failure (*n* = 16). Fifty-nine patients (88%) had a successful sETV. Eight patients failed 11 ± 8 months following surgery. Reasons for failure included obstruction of the stent, reabsorption insufficiency, and CSF leak (*n* = 2 each), and massive hygroma and tumor spread (*n* = 1 each). Complications included subdural hygroma (*n* = 4), CSF leak (*n* = 2), and stent malposition (*n* = 1). There were no complications associated with two stent removals.

**Conclusion:**

Stented ETV appears to be feasible and safe. It may be indicated in selected cases such as patients with prior ETV failure, or as a primary treatment in cases with anatomical alterations caused by tumors or thickened tuber cinereum. Future investigations are needed to further elucidate its role in non-communicating hydrocephalus.

## Introduction

Hydrocephalus is characterized by an excessive accumulation of cerebrospinal fluid (CSF) in the ventricular system, leading to an increase in intracranial pressure [1]. Endoscopic third ventriculostomy (ETV) is an effective treatment for non-communicating hydrocephalus (HCP), by creating an alternative pathway between the ventricular compartment and the subarachnoid space (SAS). Careful patient selection is critical, as patient age, etiology of hydrocephalus, and previous shunting have been shown to influence ETV success rates [2].

ETV failures fall into two main categories:

1. Early failure is when the ETV does not adequately treat the hydrocephalus. This may happen when there is an absorptive component, when the Lilliquist membrane is not opened, or in case of more distal obstruction, when the CSF does not reach the convexity SAS (such as following SAH or meningitis).
2. Late failure (usually at least 3 months after surgery) occurs when the ETV stoma occludes [3]. Late ETV failure may occur even several years following the procedure and may be life-threatening; thus the importance in avoiding its occurrence [4, 5].

Reported mechanisms of stoma closure include the following:

- Scarring or gliosis of the stoma, causing the floor of the 3rd ventricle to appear intact either by ventriculoscopy or imaging.
- Redundant tissue, whether within the floor of the 3rd ventricle, or the membrane of Lilliquist, which are associated with o closer apposition of the stoma edges.
- Arachnoid webbing [6].
- Local compression is secondary to a progressive adjacent tumor.
- Secondary to a thickened tuber cinereum.

Stoma closure can sometimes be linked to intraoperative technical factors, such as fenestration by monopolar cautery, limited size of the stoma, bleeding during the procedure, or local tumor growth.[7]

Redo ETV following prior ETV failure has success rates of 65–70% [8]. Higher rates of success can be achieved if the interval to failure is greater than 6 months since the original ETV, associated with 90% success [6]. Finally, failing ETV may be treated by adding a CSF diverting shunt [9].

The technique of placing a stent through the stoma has been described yet is not commonly used [10, 11]. It aims to place a silicone catheter tip in the prepontine cistern and has perforation holes at the tip as well as in the ventricles in order to enable a sustainable flow from internal towards external CSF spaces. Theoretically, in cases with presumably higher rates for reclosure of the stoma, a stent may reduce this risk.

There are some potential caveats with leaving a stent through the stoma. First, placing a stent may lead to blindly manipulating small perforants in the interpeduncular and prepontine cisterns, and lead to vascular injury or injury to CN3. Second, during stent fixation at the extracranial region, the catheter tip may migrate to the 3rd ventricle. Third, in case of catheter blockage or infection, it may need to be removed, potentially risking injury to the adjacent neurovascular structures. Another potential problem is a subdural hygroma, secondary to a maintained tract between the ventricle and the subdural space [12].

The primary goal of this study is to describe the indications for leaving a stent in place during an ETV and report the success rate in these cases. The secondary goal is to evaluate the safety (short and long-term) following stent placement.

## Methods

Following IRB approval, data was retrospectively collected from four different centers. The inclusion criteria were children and adults who had non-communicating hydrocephalus and underwent ETV with a stent placed through the ETV stoma (sETV). The exclusion criteria are complex hydrocephalus (multi-compartmental), presence of functioning shunt, or lack of any follow-up. Collected data included demographics, hydrocephalus etiology, history of shunt implantation, prior ETV, any other prior surgery, age at the sETV procedure, and indication for stented ETV. Details about the sETV surgery included the date of surgery, age at intervention, time between diagnosis of HCP to the surgery, and surgical complications. Surgical and clinical outcomes were expressed by clinical evaluation during follow-up, radiological follow-up, and any need for further surgeries.

## Surgical technique

**Fig. 1:**
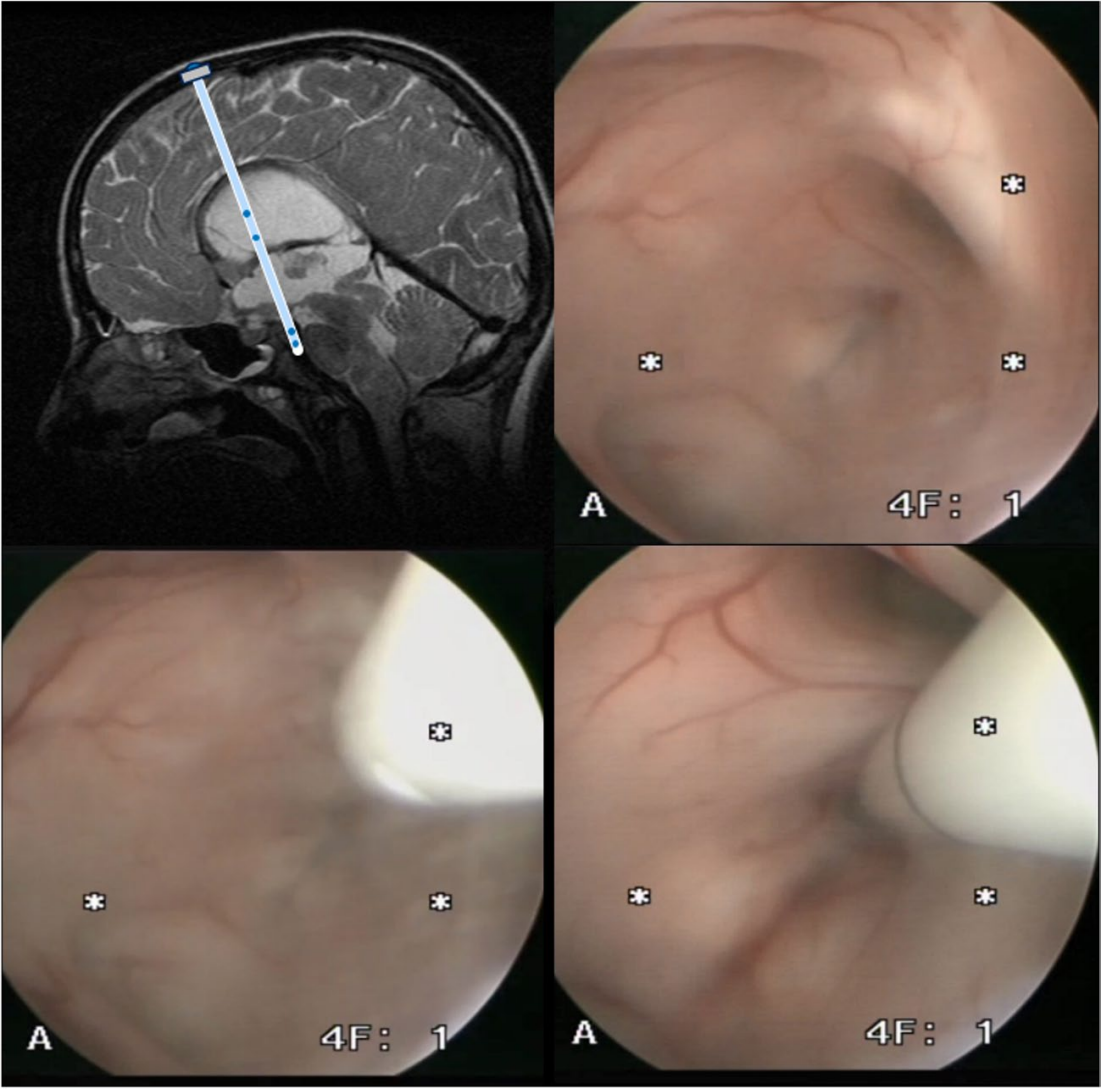
The technique of stented ETV warrants precise planning or even navigation to place the burr hole in optimal alignment with the trajectory of the prepontine cistern parallel to the clivus as well as to the basilary artery and through the floor of the 3rd ventricle as well a foramen of Monro. The aim is to place the catheter tip into the prepontine cistern with perforation holes at the tip and additionally place perforations at the lateral ventricle level (upper left). With the endoscope view positioned in the third ventricle, you can appreciate the floor of the 3rd ventricle (upper right). If the anatomy is distorted, e.g., in tumor disease navigation is warranted to identify the point of entry towards the prepontine cistern. The catheter is guided parallel to the endoscope optic (e.g., MINOP optic can be detached from the working channel, lower left). Under endoscopic view control, the tip of the catheter will be placed through the floor of the 3rd ventricle into the prepontine cistern with a penetration depth of 2.5 cm (lower right)

Following an ETV, a proximal shunt catheter with additional more proximal holes was advanced in parallel to the endoscope (Fig. 1). Once in the lateral ventricle, the catheter was advanced under vision through the ETV stoma, so that it transverses the tuber cinereum, with the tip in the subarachnoid space. The endoscope was removed, and the catheter was either anchored to the dura, or to an Ommaya reservoir (a burr hole designed, or a side inlet designed).

## Statistics

As this is a small group, only descriptive data and basic analysis (mean ± SD) are reported.

## Results

From four centers, 67 patients were included in this study, 34 males and 33 females. Primary hydrocephalus etiology included aqueductal stenosis (*n* = 19), tumor (*n* = 32), post IVH (*n* = 3), post-infection (*n* = 1), cysts (*n* = 3), post-MMC surgery (*n* = 2), tectal AVM (*n* = 1), and unknown etiologies (*n* = 6). The locations of obstruction were aqueduct of Sylvius (*n* = 36), fourth ventricle outlets (*n* = 6), third ventricle (*n* = 18), prepontine (*n* = 2), and others (*n* = 5).

Age at sETV intervention was 22.2 ± 21.1 years (range 0.1 months to 77 years old). There were 43 children (less than 18 years of age), and 24 adults. Forty patients underwent a primary sETV (with no prior surgeries treating hydrocephalus). Twenty-seven underwent secondary sETV following a prior shunt failure (*n* = 11), prior ETV failure (*n* = 15), or failure of both a shunt and ETV (*n* = 1).

**Fig. 2:**
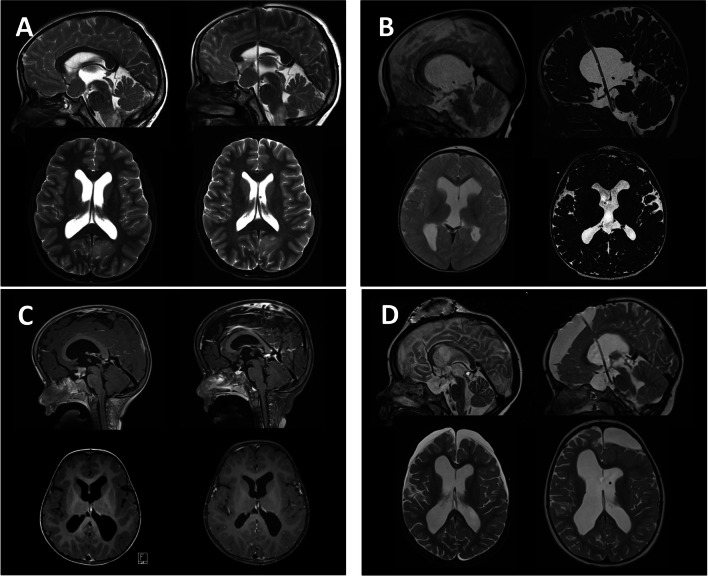
Representative cases of stented ETV in various conditions. **A** 10-year-old boy with neurofibromatosis type 1 and a bilateral optic nerve glioma involving the chiasm and aqueductal stenosis. The catheter stent was placed, resulting in decrease in ventricular width and more balanced subarachnoid spaces. B: 1.5-year-old girl with congenital aqueductal stenosis with consecutive triventricular hydrocephalus and anatomical alterations at the level of the 3rd ventricular floor which leads to a smaller membrane. A stent was placed for a sustainable communication between internal and external CSF spaces. **C** 2.5-year-old boy with intrinsic chiasmatic tumor involving the floor of the 3rd ventricle and aqueductal stenosis with ventricular enlargement. Stent placement led to a ventricular size decrease and LGG chemotherapy resulted in a partial response of the tumor disease. **D** 2-year-old boy with disseminated tumor disease and triventricular hydrocephalus. After interhemispheric transcallosal tumor debulking hydrocephalus condition was not resolved and CSF collection was observed. A stented ETV led to the resolution of the CSF collection; however, in the long run, metastatic condition led to malabsorptive hydrocephalus, and a shunt was necessary to implant

Indications for sETV included local tumor (*n* = 15), thickened 3rd ventricular floor (*n* = 23), prior ETV failure (*n* = 16), young age at the procedure (*n* = 4), redundant tuber cinereum (*n* = 1), low pressure hydrocephalus (*n* = 1), prior tumor surgery in the vicinity to stoma region (*n* = 1), and unknown reasons (*n* = 6) (Fig. 2). Two different techniques were used in this cohort: In 55 patients the transventricular stent was anchored with an Ommaya reservoir, while in 12 the stent was anchored to the dura without any reservoir. The follow-up period was 43 ± 40 months, (range 3 weeks–168 months).

## Complications

Seven patients (10%) had complications, none of which were major. Four had a subdural hygroma (1 was treated by a shunt), 1 had a stent malposition (treated conservatively), and 2 had CSF leaks (treated with a shunt). Overall, 2 stents were removed, 1 due to CSF leak, and 1 during a subsequent tumor surgery following patency verification of the stoma. Two stents were converted to shunts due to sETV failure. No complications were associated with stent removal.

## Outcome

Eight patients (12%) failed the sETV with consecutive shunt implantation (ventriculo-peritoneal shunt, *n* = 7, and subdural-peritoneal shunt, *n* = 1). Four (10%) of the primary sETV failed. and 4 (15%) of secondary sETV failed (2 of 15 with a prior ETV failure, and 2 of 11 with a prior shunt failure). The time from sETV procedure to failure was 11 ± 8 months (range 4–30 months). Reasons for failure included obstruction of the stent (*n* = 2), massive hygroma (*n* = 1), diffuse tumor spread (*n* = 1), CSF reabsorption insufficiency (*n* = 2), and CSF leak (*n* = 2).

## Discussion

In the current series, which represents the largest to date on the use of stents during ETV, we have shown that the technique appears to be feasible and rather safe. The overall success rate of stented ETV was relatively high (88%), with no major morbidity.

While the ETV success score (ETVSS) estimates preoperative predictive success of an ETV, other factors may affect ETV success, such as a thickened or redundant third ventricular floor. The presence of a tumor adjacent to the ETV location may also lead to closure by local compression. Additionally, a prior ETV failure may also indicate a potential higher risk for redo ETV failure, although this point is debatable. In this context, the relative high success in our series appears to be especially striking since most of the cases were associated with any higher risk of ETV failure.

Thus, a stented ETV was added to avoid ETV closure due to local scarring or tumor compression [11]. Note that these are all *selected* cases, each with specific nuances and considerations, which do not represent the classical variables analyzed for the ETVSS. Therefore, we cannot compare the outcomes of this series to the outcomes anticipated by ETVSS. However, all patients included in this study suffered from non-communicating hydrocephalus, and the ETV was indicated to reestablish communication between internal and external CSF spaces. Nevertheless, all patients were judged not to be treated successfully by a regular ETV alone. We postulate that risk estimation in this cohort may need new evaluation for success using the sETV technique.

When considering the 12% failures, we acknowledge that the stent implantation may also have limitations leading to failure, such as obstruction or infection. Another complication—subdural hygromas—has already been described when using ventricular catheters and Ommayas following ETV in children, possibly caused by maintaining a ventricular subdural tract along the catheter [12]. This may be avoidable by better sealing the stent tract and avoiding leakage of CSF.

## Limitations

This is a retrospective study including a limited number of patients, representing a heterogeneous group of cases, including children and adults. Despite the inclusion of children and adults, we believe that the considerations regarding the placement of a stent are similar, and can be implied in all age groups. The data is limited since not all indications for leaving a stent were reported. Our cohort has a short follow-up, possibly leading to under-diagnosis of late ETV failure. Thus, the described experience from four centers is still limited; however, the data contributes to better indicating the technique in respective cases.

## Conclusions

Stented ETV is a feasible and safe technique, which may be indicated in selected cases, such as patients with prior ETV failure related to local scarring, or as a primary treatment, especially when there is an adjacent tumor which may grow and obstruct the stoma, or a thickened or redundant tuber cinereum. Future data collection, preferably in a prospective manner will be warranted to draw stronger conclusions in terms of optimal technique and indication.

## Data Availability

The data that support the findings of this study are available on request from the corresponding author.
